# Identification of new prognostic molecular markers in glioblastoma: a single-center retrospective study

**DOI:** 10.1093/oncolo/oyag095

**Published:** 2026-03-16

**Authors:** Adèle Meola, Bertille Segier, Aurore Siegfried, Amélie Lusque, Solène Evrard, Damien Pouessel, Delphine Dghayem, Franck-Emmanuel Roux, Sandra Soares, Maïlys Guillaumey, Ilfad Blazevic, Elizabeth Cohen-Jonathan Moyal, Delphine Larrieu

**Affiliations:** Department of Oncology, Univ Toulouse, Oncopole Claudius Regaud, IUCT-Oncopole, Toulouse, 31059, France; INSERM U1037, Cancer Research Center of Toulouse (CRCT), Toulouse, 31024, France; Biostatistics and Health Data Science Unit, Univ Toulouse, Oncopole Claudius Regaud, IUCT-Oncopole, Toulouse, 31059, France; INSERM U1037, Cancer Research Center of Toulouse (CRCT), Toulouse, 31024, France; Department of Pathology, Univ Toulouse, Oncopole Claudius Regaud, IUCT-Oncopole, Toulouse, 31059, France; Biostatistics and Health Data Science Unit, Univ Toulouse, Oncopole Claudius Regaud, IUCT-Oncopole, Toulouse, 31059, France; INSERM U1037, Cancer Research Center of Toulouse (CRCT), Toulouse, 31024, France; Department of Pathology, Univ Toulouse, Oncopole Claudius Regaud, IUCT-Oncopole, Toulouse, 31059, France; Department of Oncology, Univ Toulouse, Oncopole Claudius Regaud, IUCT-Oncopole, Toulouse, 31059, France; Department of Neuroradiology, Hopital Pierre Paul Riquet, CHU Purpan, Toulouse, 31059, France; Department of Neurosurgery, Hopital Pierre Paul Riquet, CHU Purpan, Toulouse, 31059, France; Department of Oncology, Univ Toulouse, Oncopole Claudius Regaud, IUCT-Oncopole, Toulouse, 31059, France; Department of Oncology, Univ Toulouse, Oncopole Claudius Regaud, IUCT-Oncopole, Toulouse, 31059, France; Department of Oncology, Univ Toulouse, Oncopole Claudius Regaud, IUCT-Oncopole, Toulouse, 31059, France; INSERM U1037, Cancer Research Center of Toulouse (CRCT), Toulouse, 31024, France; Department of Radiotherapy, Univ Toulouse, Oncopole Claudius Regaud, IUCT-Oncopole, Toulouse, 31059, France; Department of Oncology, Univ Toulouse, Oncopole Claudius Regaud, IUCT-Oncopole, Toulouse, 31059, France

**Keywords:** glioblastoma, prognostic factor, molecular, EGFR, survival analysis

## Abstract

**Background:**

Glioblastoma (GBM) is the most common and aggressive primary CNS tumor in adults and carries a poor prognosis. Although molecular classification has advanced, patient outcomes remain variable. Next-generation sequencing (NGS) can reveal genomic alterations that, while not yet treatment-guiding, offer prognostic and biological insight. The study aimed to investigate the association between molecular alterations in primary GBM tumors and patient survival outcomes.

**Methods:**

This study analyzed medical records of consecutive GBM patients treated between November 1, 2018 and December 31, 2021 at a comprehensive cancer center, for whom NGS data from the tumor was available. Survival analyses were performed according to clinical, radiological, therapeutic, and molecular data.

**Results:**

Of 199 patients, 38 were excluded: 15 for recurrence-only data and 23 for incomplete molecular data after first progression. In the survival analysis of the cohort (*n* = 161) adjusted on surgical resection, MGMT promoter methylation status, number of alterations, age, tumor localization, MIB1 and performance status, EGFR substitutions were significantly associated with increased overall survival (OS) (HR = 0.43 [0.26; 0.72], *P* = .001), while CDKN2A/B loss and TP53 substitutions were associated with decreased OS (HR = 1.75 [1.08; 2.84], *P* = .023 and HR = 1.69 [1.04; 2.74], *P* = .034 respectively). NOTCH1 substitutions showed a trend towards improved progression-free survival (PFS) (HR = 0.55 [0.30; 1.01], *P* = .054).

**Conclusion:**

We identified new prognostic markers in GBM, showing for the first time that EGFR substitutions improve OS. Validation in external cohorts and preclinical studies is needed.

Implications for practice Glioblastoma currently has only a few recognized prognostic factors, particularly at the molecular level. MGMT promoter methylation remains the only well-established predictive marker of chemotherapy response, yet patient survival and therapeutic outcomes vary widely. In this study, we report the identification of novel prognostic molecular markers derived from a large molecular dataset generated through high-throughput sequencing. EGFR substitutions were associated with favorable prognosis, whereas TP53 substitutions and CDKN2A/B loss correlated with poorer overall survival.

Despite the limitations of a single-center retrospective study and the need for further mechanistic research, our findings may inform future molecularly guided clinical decision-making in progressive glioblastoma.

## Introduction

Glioblastoma (GBM) is currently the most common and aggressive primary central nervous system (CNS) tumor in adults, with a very poor prognosis. Since 2016, histopathological diagnosis has been completed by the identification of molecular markers, leading to a new classification of CNS tumors. In addition to histopathological criteria (increased cellular density, nuclear atypia, mitosis, vascular proliferation and necrosis), the 2021 update of the World Health Organization (WHO) classification also defines GBM by glioma cells without *isocitrate dehydrogenase* (*IDH)* mutation, and with the presence of one of the following molecular alterations: *EGFR* amplification, *TERT* promoter mutation and/or gain of chromosome 7 and loss of chromosome 10.^1–3^ Despite these molecular advances in classification, heterogeneity persists in outcomes and survival among GBM patients.^4^^,^^5^

Standard first-line treatment of GBM has remained unchanged for almost 20 years (Stupp protocol),^6^ until the advent of Tumor Treating Fields therapy.^7^ No standardized treatment is recommended for relapsed/refractory GBM, and their management remains challenging.

Outside GBM, the better understanding of signaling pathways in cancer cells has led in the last decade to the development of modern therapies based on molecular characteristics such as targeted therapy or immunotherapy.^8^ This precision medicine has significantly improved the prognosis of cancers that were previously associated with poor outcomes (eg, metastatic melanoma, non-small lung cell cancer) and opened a new treatment era.^9^^,^^10^

**Figure 1.**
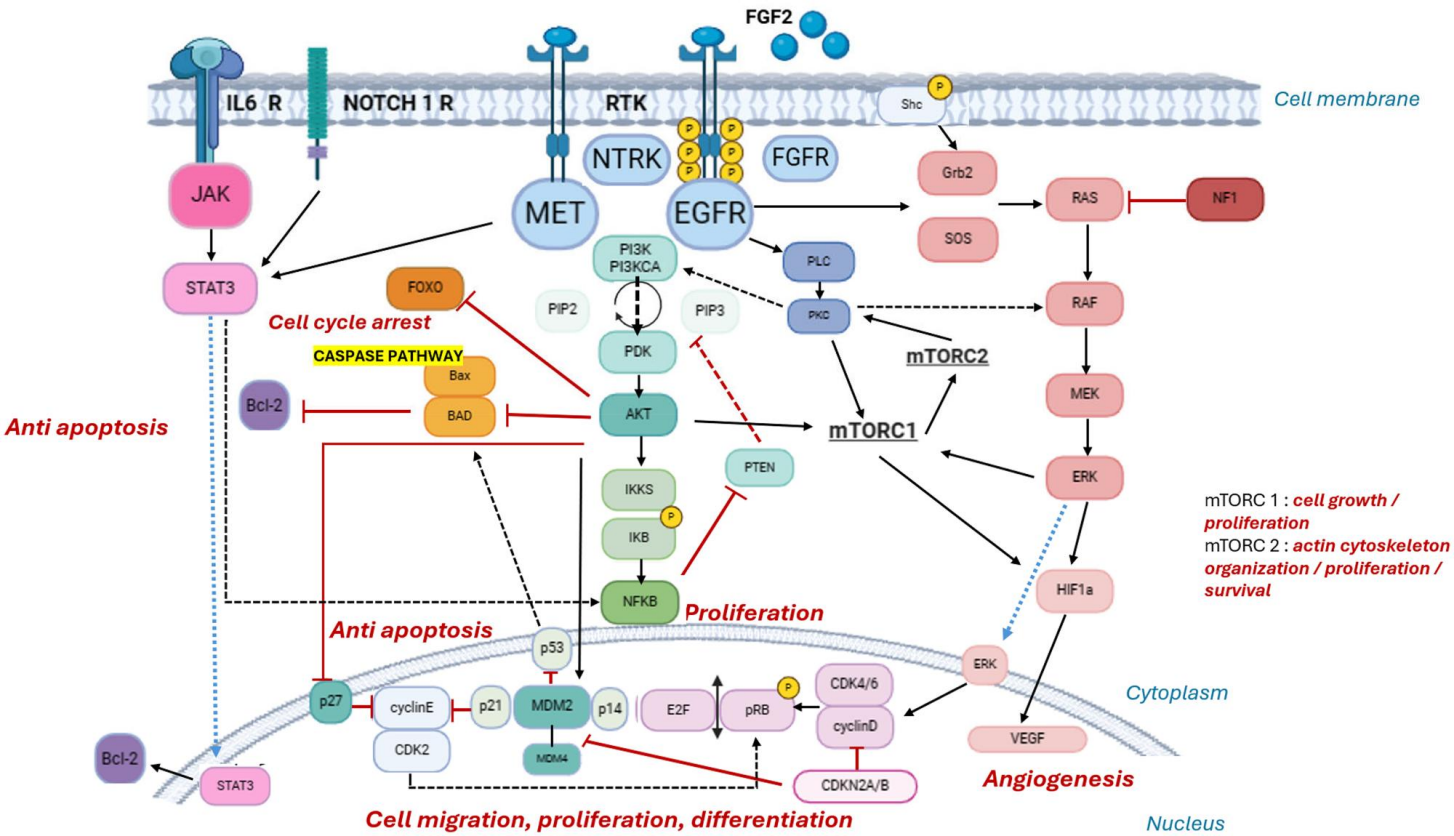
Major signaling pathways altered in glioblastoma. Black arrows indicate activation and red arrows indicate inhibition. Blue arrows indicate protein transfer from cytoplasm to nucleus. Black and red dotted lines indicate secondary pathways of activity. Note: MDM2 inhibition of P53 is both intra-nuclear and intra-cytoplasmic. Cyclin D/CDK4-6 and cyclin E/CDK2 complexes are responsible for cell transition to G1 phase and centrosome duplication. p21 and p27 act as tumor suppressors by blocking cyclins. AKT inhibits the normally pro-apoptotic caspase 9/3 pathway. Produced using Biorender.com software.

Main signaling pathways are activated in GBM cells, including PI3K/AKT/mTOR and Ras/Raf/MEK/ERK (Figure 1). They promote cell progression, growth and survival.^11^ However, to date, clinical efficacy has only been suggested in highly selective and specific situations.^12^ In the majority of patients, targeting pathways commonly altered in GBM has not yet demonstrated clinical efficacy.^13–16^ In addition to blood-brain barrier, redundant compensatory mechanisms and insufficient target coverage are some of the hypotheses put forward to explain these failures.

During the last decade, companion tests based on next-generation sequencing (NGS) have been developed to detect diagnostic or theragnostic relevant genomic alterations in cancer cells, including GBM. Such broad-spectrum tests reveal numerous undescribed molecular alterations or of unknown significance. These latter alterations may not lead to disease classification nor targeted therapy or treatment optimization. However, they exhibit high scientific interest regarding GBM prognosis and pathophysiology.

In this study, we aimed to highlight the association between molecular alterations identified on the initial GBM tumor and overall survival (OS) of patients. Our secondary objectives were to assess their impact on progression-free survival (PFS), and to describe the management and evolution of the patients who received genomic-guided therapy.

## Methods

This study was conducted after the project was declared to the HDH registry to comply with CNIL regulations. A waiver was granted for informed consent due to the retrospective nature of the study.

### Cohort selection

All consecutive adult patients (≥18-years-old) with a pathological diagnosis of *IDH* wildtype (*IDHwt*) GBM, WHO grade 4 (2021 classification), treated at IUCT Oncopole, Toulouse from November 1, 2018 to December 31, 2021, and had molecular information through a molecular test using NGS were screened. Patients were included if they had a Performance Status (PS) < 3, or a PS of 3 that was exclusively related to a neurological deficit (Supplementary Appendix 1—see online supplementary material).

**Figure 2.**
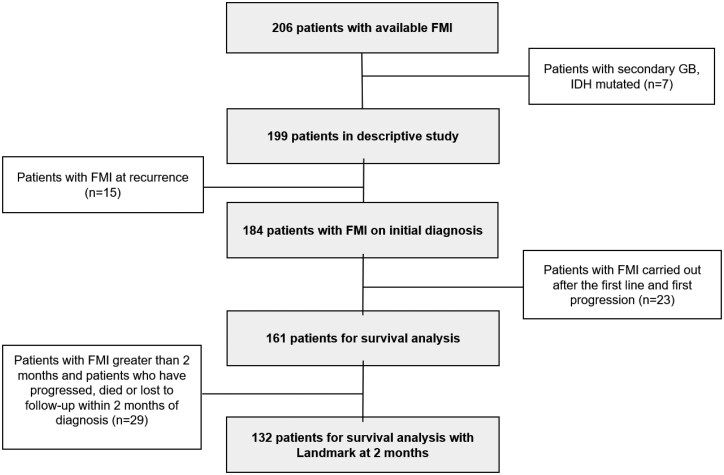
Flow-chart of the study.

Exclusion criteria were: (i) *IDH* mutant glioma, (ii) molecular test using NGS results on tumor recurrence samples, (iii) opposition to the study (Figure 2).

### Data collection

A review of the patients’ medical and clinical records was carried out. Each patient was reviewed and clinical data, including gender, age, PS, topography of the lesion, histomolecular diagnosis, extent of resection, routinely performed biomarkers like *O6-methylguanine-DNA methyltransferase* (*MGMT*) promoter methylation status, first-line treatment, date of first progression, number of therapeutic lines performed, and date of last news and/or death were collected retrospectively.

The extent of resection was determined by early postoperative MRI (<72 h), classified as partial (mesurable contrast enhancement with a maximum diameter exceeding 1 cm in at least one of the three spatial planes) or subtotal/macroscopically complete. Gross total resection was defined as the absence of measurable contrast enhancement, while subtotal resection was defined as measurable contrast enhancement with a maximum diameter of 1 cm or less in all three spatial planes.

For biopsies, early postoperative CT defined a third surgery group.

For first-line therapy, radiotherapy dose (grays—Gy), timing, and combination with temozolomide (TMZ) and/or bevacizumab (BVZ) were recorded. The number of adjuvant cycles was noted: TMZ (5 days/month, 100-200 mg/m^2^) and BVZ (two injections, 15-21 days apart, 5 mg/kg/week). TTFields use was also documented.

Molecular testing, targeted therapies, treatment lines, and progression data were collected.

Progression was defined by the first MRI showing recurrence (*modified RANO criteria*). In cases of uncertainty between true progression and pseudo-progression, a follow-up MRI was performed one month later; if progression was confirmed, the date of the initial MRI was recorded as the date of progression.

### Pathology and molecular data

Diagnosis followed the 2021 WHO classification; if uncertain, cases were reclassified by a RENOCLIP expert *(RENOCLIP-LOC, INCa-certified network for rare CNS tumors)*.

*IDH1/2* mutations were analyzed by NGS. *MGMT* promoter methylation was assessed by pyrosequencing or PCR, when available.

All tumors were sequenced using FoundationOne^®^CDx (FMI, from *Foundation Medicine Inc., Cambridge, MA 02141, USA*), a 324-gene NGS panel detecting substitutions, insertions/deletions (*indels*) and copy number alterations (CNAs), as well as certain rearrangements within selected genes.^8^^,^^17^ It also analyzes genomic signatures such as microsatellite instability (MSI) and tumor mutational load (TMB) from DNA isolated from Formalin-Fixed Paraffin-Embedded (FFPE) tumor tissue. Targeted genes are listed in Supplementary Appendix 2 (see online supplementary material).

Gene names followed HUGO nomenclature (italics for genes, roman for proteins).

Each alteration was classified as known or variant of uncertain significance (VUS).

Specific codon details were unavailable for partial deletions/amplifications.

### Statistical analysis

Patients’ characteristics were summarized using median and range [min-max] for continuous variables and number and percentage for qualitative variables. The number of missing values were described for all variables.

Survival rates were estimated using Kaplan Meier method with their 95% Confidence Interval (95% CI). OS and PFS were calculated from initial diagnosis. First event definition was death from any cause for OS and progression or death for PFS. Patients who were still alive were censored at their date of last contact.

Univariable and multivariable analyses were performed using Cox model with left truncation to consider selection bias induced by the time between initial diagnosis and FMI.

An initial multivariable analysis was performed independently for each gene, adjusting for clinical factors. Then, a general multivariable model including genes associated with 20% in first multivariable analysis, number of alterations and clinical factors was made.

A Landmark approach at 2 months, excluding patients who died, recurred or were lost to follow-up before 2 months and patients with FMI greater than 2 months was used in addition, to check the robustness of the general multivariable model results.

OS on targeted therapy was described and defined as the time between the date of initiation of targeted therapy and the date of death from any cause. Patients who were still alive, including those with ongoing targeted therapy, were censored at the last reported date.

Statistical tests were two-sided and *P*-value <.05 was considered significant. All statistical analyses were conducted using STATA version 18 software.

## Results

Of 206 GBM patients with FMI, 7 with IDH mutations were excluded, leaving 199 *IDHwt* cases. After excluding 15 with FMI at recurrence, 184 were analyzed for mutations. An additional 23 with FMI performed only after progression were excluded from survival analyses, yielding a final cohort of 161 patients (Figure 2).

### Patient characteristics

Baseline and relevant characteristics of the whole cohort (*n* = 199) are summarized in Table 1 (median age 62 years, [23-86]). *MGMT* promoter methylation was available for 186 patients; 40% were methylated (*n* = 74) and 60% unmethylated (*n* = 112). The median MIB index was 25% [4-80]. Thirty patients (15.4%) had a biopsy, 101 (51.8%) a partial resection, and 64 (32.8%) a subtotal or complete resection. The median delay to receive FMI results was 1.3 months [0.3-50.2].

**Table 1.** Patient characteristics for cohort of 199 GBM patients.

**Population (*N* = 199)**			
**Median age at diagnosis [range]**	62 [23-86]	**MIB1 (%)** *n* = 193	25 [8-40]
**Sex**		**Median time to obtaining FMI**	1.3 [0.3-50.2]
** Male**	129 (64.8%)	**First-line therapy**	
** Female**	70 (35.2%)	***Concurrent chemoradiotherapy***	189 (95.0%)
**Performance status**		*Radiotherapy dose*	
** 0-1**	168 (84.4%)	60 Gy/30 fractions	182 (96.3%)
** 2-3**	31 (15.6%)	45 Gy/15 fractions	7 (3.7%)
**Tumor localization**		*With concurrent BEV*	
** Frontal**	97 (48.7%)	Yes	10 (5.3%)
** Other**	102 (51.3%)	No	178 (94.2%)
**Hemispheric lateralization**		Missing	1 (0.5%)
** Right**	103 (51.8%)	***TMZ post chemoradiotherapy***	
** Left**	84 (42.2%)	Yes	175 (92.6%)
** Bilateral**	12 (6%)	No	14 (7.4%)
**Extent of resection**		Median number of cycles	6 [1-18]
** Biopsy**	30 (15.0%)	***BEV post chemoradiotherapy***	
** Partial resection**	101 (50.8%)	Yes	20 (11.4%)
** Subtotal/macroscopically complete**	64 (32.2%)	No	155 (88.6%)
** Missing**	4 (2.0%)	Median number of cycles	6.5 [2-51]
**MGMT methylation status (%)**		***TMZ chemotherapy without radiotherapy***	10 (5.0%)
** Methylated**	74 (37.2%)	*With concurrent BEV*	
** Unmethylated**	112 (56.3%)	Yes	8 (80.0%)
** Missing**	13 (6.5%)	No	2 (20.0%)

%: proportion.

[ ]: extrem values.

A total of 189 patients (95.0%) received concomitant chemoradiotherapy combining radiotherapy and TMZ. Of the 189 patients, 182 patients (96.3%) were treated by concomitant chemoradiotherapy according to the standard Stupp protocol and received a radiation dose of 60 Gy.^6^ Seven patients (3.7%) received a total of 45 Gy according to the Perry protocol.^18^ BVZ was administered alongside chemoradiotherapy in 10 patients (5.3%) to enhance tolerance to radiotherapy for large lesions.^19^ A total of 175 patients (92.6%) received subsequent cycles of TMZ, with a median of 6 cycles [1-18]. Additionally, 20 patients received BVZ treatment with a median of 6.5 cycles. Ten patients (5.0%) received chemotherapy (TMZ) alone as first-line treatment, of which 8 received BVZ concomitantly. The median total number of treatment lines was 3 [1-7].

### Reported molecular alterations

The frequency of the main molecular alterations found in the 184 patients included in the descriptive mutational analysis is detailed in Supplementary Appendix 3 (see online supplementary material).

The most common genetic alterations found were non-truncating mutations: substitutions and insertion-deletions (indels).

It is important to note that the same patient could have multiple molecular alterations targeting the same gene. Similarly, the same type of alteration on the same gene could occur multiple times in the same patient (eg, *EGFR* heterozygous deletion).

Median number of molecular alterations per patient reported on the FMI was 13 [4-291]. The most common alterations included *TERT* promoter substitutions (*n* = 163, 88.6%), loss of *CDKN2A* (*n* = 121, 65.8%), loss of *CDKN2B* (*n* = 113, 61.4%), *PTEN* alteration (*n* = 118, 64.1%), *MTAP* loss (*n* = 95, 51.6%), *EGFR* amplification (*n* = 86, 46.7%), *EGFR* heterozygous deletion (*n* = 57, 31.0%), *EGFR* substitution (*n* = 54, 29.3%), *TP53* substitutions (*n* = 39, 21.2%), *PDGFRA* amplification (*n* = 16, 8.7%), *KMT2D* substitutions (*n* = 24, 13.0%) and *NOTCH1* substitutions (*n* = 21, 11.4%).

Other potentially targetable molecular alterations were *POLE* substitutions (*n* = 11, 6.0%), *BRAF* V600E (*n* = 8, 4.3%), *HGF* amplification (*n* = 7, 3.8%), *NF1* loss (*n* = 5, 2.7%), *FGFR3* fusions (*n* = 4, 2.2%) and *FGFR1* fusions (*n* = 1, 0.5%).

Seven patients (3.6%) presented a high TMB (≥ 10 mutations per megabase) including 2 patients with a constitutional mutation of *POLE*.

It is important to note that the substitutions reported for *NOTCH1* were reported as VUS.

### Description of gene association

*PTEN* loss was more commonly significantly associated with *CDKN2A* loss (*P* = .020), *CDKN2B* loss (*P* = .010), *EGFR* substitution (*P* = .032), *EGFR* amplification (*P* = .001) and *EGFR* heterozygous deletion (*P* = .013). In our cohort, all *MDM2* amplification were associated with *CDK4* amplification (*P* < .0001).

### Survival data

**Figure 3.**
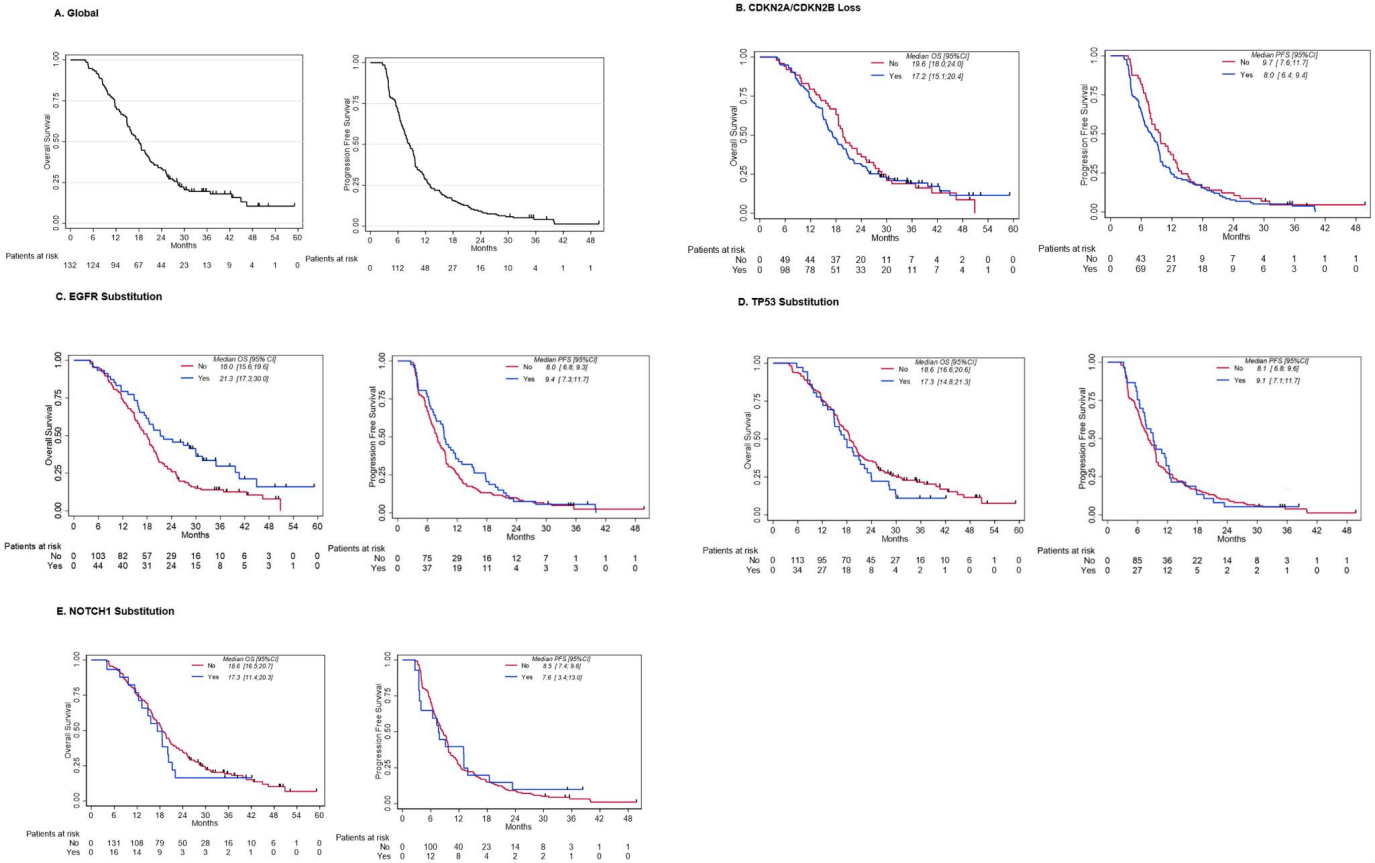
Overall survival (OS) and progression-free survival (PFS) curves: Left truncation (*n* = 161). Blue line: Yes; red line: No. The curve on the left represents overall survival (OS), and the curve on the right represents progression-free survival (PFS). OS data are shown in panel A (*n* = 161), while data according to CDKN2A/CDKN2B loss, EGFR substitution, TP53 substitution and NOTCH1 substitution are presented in panels B, C, D, and E, respectively.

After a median follow-up (*n* = 161) of 38.2 months [95% CI: 34.9; 49.3], disease progression was observed in 149 (92.5%) patients and 135 patients (83.9%) had died. The median OS was 18.6 months [16.5; 20.4] and the median PFS was 8.5 months [7.4; 9.6] (Figure 3A).

#### Univariable analysis

In univariable analysis, poorer OS was observed with PS 2-3 (HR = 1.98 [1.27-3.09], *P* = .002), while age was not significantly associated. None of these factors were significant for PFS.

Higher MIB1 was associated with reduced OS (HR = 1.01 [1.00-1.02], *P* = .022), whereas the number of molecular alterations showed no association with OS or PFS. *MGMT* promoter methylation correlated with improved OS (HR = 0.41 [0.28-0.60], *P* < .001) and PFS (HR = 0.40 [0.28-0.57], *P* < .001).

*EGFR* substitutions were linked to better OS (HR = 0.60 [0.41-0.88], *P* = .009) but not PFS. *PTEN* loss showed a trend toward improved OS (HR = 0.72 [0.51-1.02], *P* = .067) without PFS association, while *PTEN* substitutions were not significant. *CDK4* amplification tended to reduce PFS (HR = 1.58 [0.94-2.65], *P* = .082) with no OS effect.

No significant associations with OS or PFS were observed for *CDKN2A/B* loss, *TP53, KMT2D, NOTCH1, TERT* promoter, *PTPN11*, or *PIK3CA* substitutions.

The results are summarized in Figure 3 and Supplementary Appendix 4 (see online supplementary material).

#### Multivariable analysis

**Figure 4.**
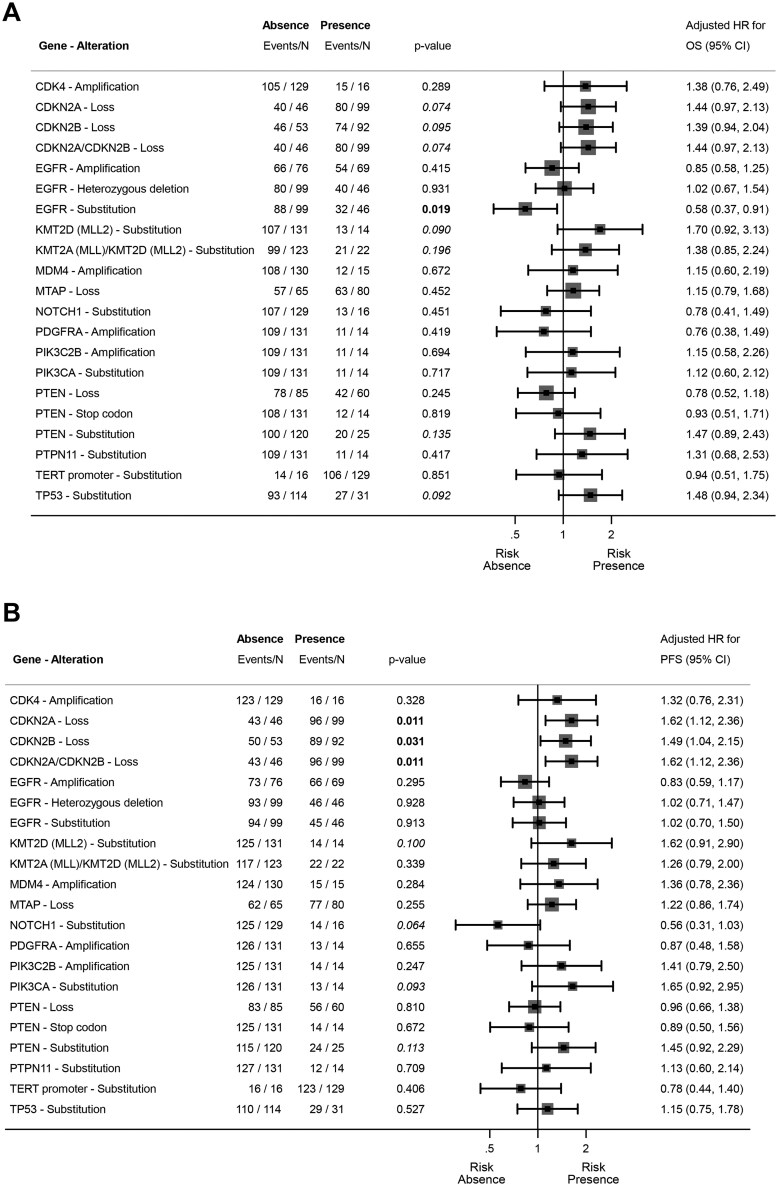
Forest plot summarizing the results of Overall survival (OS) (A) and Progression-free survival (PFS) (B) independent multivariable models for each gene. *P*-values <.05 are in bold, and *P*-values <.2 are in italics to show the selection of genes included in the general multivariable model.

OS and PFS results of the initial multivariable analysis by gene, according to clinical factors, are shown in Figure 4. OS and PFS results of the general multivariable analysis according also to the genes associated with 20% in first multivariable analysis are presented in Tables 2 and 3.

**Table 2.** OS multivariable analysis—several genes including the genes associated with 20% (Cox model with left truncation, Evt/n = 120/145).

Event	HR [95% CI]	*P*-value
***CDKN2A/CDKN2B—*loss**		
**No**	1.00	
**Yes**	1.75 [1.08; 2.84]	**.023**
***EGFR—*substitution**		
**No**	1.00	
**Yes**	0.43 [0.26; 0.72]	**.001**
***KMT2D (MLL2)*—substitution**		
**No**	1.00	
**Yes**	1.49 [0.80; 2.77]	.212
***PTEN—*substitution**		
**No**	1.00	
**Yes**	1.08 [0.64; 1.81]	.769
***TP53*—substitution**		
**No**	1.00	
**Yes**	1.69 [1.04; 2.74]	**.034**
**Age at diagnosis**		
**<60**	1.00	
**≥60**	1.73 [1.10; 2.71]	**.018**
***Performance* status**		
**0-1**	1.00	
**2-3**	1.19 [0.68; 2.10]	.541
**Extent of resection**		
**Biopsy**	1.00	
**Partial resection**	0.62 [0.34; 1.12]	.115
**Subtotal/macroscopically complete**	0.43 [0.23; 0.81]	**.009**
**MGMT promoter methylation status (%)**		
**Unmethylated**	1.00	
**Methylated**	0.35 [0.22; 0.55]	**<.001**
**Tumor localization**		
**Other**	1.00	
**Frontal**	1.23 [0.82; 1.85]	.309
**Number of alterations**	1.05 [1.00; 1.10]	**.048**
**MIB1 (%)**	1.01 [1.00; 1.02]	.114

The values in bold are statistically significant *p*-values (p < 0.05).

**Table 3.** PFS multivariable analysis—several genes including the genes associated with 20% (Cox model with left truncation, Evt/n = 139/145).

Event	HR [95% CI]	*P*-value
***CDKN2A/CDKN2B—*loss**		
**No**	1.00	
**Yes**	1.62 [1.05; 2.52]	**.031**
***NOTCH1*—substitution**		
**No**	1.00	
**Yes**	0.55 [0.30; 1; 01]	*.054*
***KMT2D (MLL2*)—substitution**		
**No**	1.00	
**Yes**	1.36 [0.74; 2.50]	.316
***PTEN—*substitution**		
**No**	1.00	
**Yes**	1.24 [0.78; 2.00]	.366
***PIK3CA—*substitution**		
**No**	1.00	
**Yes**	1.79 [0.96; 3.34]	*.068*
**Age at diagnosis**		
**<60**	1.00	
**≥60**	1.31 [0.86; 1.97]	.206
***Performance* status**		
**0-1**	1.00	
**2-3**	0.98 [0.59; 1.63]	.953
**Extent of resection**		
**Biopsy**	1.00	
**Partial resection**	1.06 [0.61; 1.83]	.844
**Subtotal/macroscopically complete**	0.90 [0.49; 1.64]	.723
**MGMT promoter methylation status (%)**		
**Unmethylated**	1.00	
**Methylated**	0.31 [0.21; 0.46]	**<.001**
**Tumor localization**		
**Other**	1.00	
**Frontal**	1.27 [0.87; 1.86]	.224
**Number of alterations**	1.02 [0.97; 1.06]	.499
**MIB1 (%)**	1.01 [1.00; 1.02]	.112

The values in bold are statistically significant p-values (*p* < 0.05).

The values in italics are non-significant p-values that are close to the significance threshold of 0.05.

##### Identification of molecular factors influencing survival

An increasing number of genomic alterations demonstrated a shorter OS (HR = 1.05 [95% CI: 1.00-1.10], *P* = .048), and was also significantly associated with worse OS in the Landmark analysis (HR = 1.10 [95% CI: 1.03-1.19], *P* = .008).

In the general multivariable model, *MGMT* promoter methylation remained significantly associated with better OS (HR = 0.35 [0.22; 0.55], *P* < .001) and PFS (HR = 0. 31 [0.21; 0.46], *P* < .001). The loss of *CDKN2A* and/or *CDKN2B* was associated with a poor prognosis in OS (HR = 1.75 [1.08; 2.84], *P* = .023) and PFS (HR = 1.62 [1.05; 2.52], *P* = .031). EGFR substitutions were significantly associated with a better OS (HR = 0.43 [0.26; 0.72], *P* = .001).

The presence of *TP53* substitutions was associated with decreased OS (HR = 1.69 [1.04; 2.74], *P* = .034). No effect on PFS was detected.

*NOTCH1* substitutions tended to have a better prognosis in PFS (HR = 0.55 [0.30; 1.01], *P* = .054). It is important to note that all reported *NOTCH1* substitutions were classified as VUS.

Despite a less pronounced trend, *PIK3CA* substitutions seemed to be associated with shorter PFS (HR = 1.79 [0.96; 3.34], *P* = .068).

##### Other factors influencing survival

An age of 60 years or older was associated with significantly poorer OS (HR = 1.73 [95% CI: 1.10-2.71], *P* = .018). A PS scored 2-3 was found to be non-significant on OS (*P* = .542) and PFS (*P* = .953) (Tables 2 and 3).

However, it is important to note that the initial surgical management significantly influenced the OS: the more extensive the surgery, the better the overall prognosis. Compared to biopsy, partial resection was not statistically significant (HR = 0.62 [95% CI: 0.34-1.12], *P* = .115), while subtotal or complete resection was (HR = 0.43 [95% CI: 0.23-0.81], *P* = .009). No association was observed with PFS.

Despite some slights differences, Landmark analysis at 2 months confirmed the results of the main analysis (Supplementary Appendix 5A and B—see online supplementary material).

#### Subgroup analyses

In the final survival analysis cohort (*n* = 161), 77 patients had *EGFR* amplification and 50 had at least one *EGFR* substitution; only 12 (24%) showed a substitution without amplification, consistent with prior reports.

A quarter of *EGFR* substitutions associated with better OS were classified as VUS. Median OS for the two most frequent substitutions, A289 and G598, was 16.1 months [9.0-31.6] and 15.5 months [4.0–not reached], respectively—less favorable than the overall cohort. The *EGFR* R108 subgroup was too small for survival analysis. Among patients with *MGMT* promoter methylation (*n* = 61), median OS and PFS were 25.4 months [19.6-39.7] and 12.4 months [9.8-15.6], respectively. A trend toward poorer OS was observed with TP53 substitution (HR = 1.91 [0.98-3.60], *P* = .056).

## Discussion

The main objective of this study was to identify new prognostic molecular markers in GBM based on the molecular screening conducted on the initial tumor lesion.

Studies have already been conducted to identify prognostic signatures such as the ATE score, which combines *MGMT* promoter methylation with specific genetic markers (CHEK1, GPR17, IGF2BP3, MTHFD1L, PTRH2, SOX11, S100A9, and TFRC). However, it is not widely applicable in clinical practice.^20^

Compared to the latest series of GBM published in the literature, our cohort appears to be homogeneous and comparable in terms of clinical and therapeutic characteristics. The median OS was 18.6 months [16.5; 20.4] and the median PFS was 8.5 months [7.4; 9.6] in our study. These results are superior to those reported in historical cohorts.^21^^,^^22^ They are comparable to those of more recent series, such as the one published by Lim Fat et al. (OS of 20.1 months and PFS of 9.1 months).^23^

Focusing on clinical criteria, by multivariable analysis, an age older than 60 years seems to be predictive of a poor prognosis as has been widely reported.^24^^,^^25^ Our findings suggest that *PS* is not an independent predictor of survival, consistent with prior studies showing no association between KPS and outcomes after initial resection.^26^ These results argue against basing therapeutic decisions, including ICU admission, solely on PS in patients with primary brain tumors.^27^^,^^28^

Beyond the inherent limitations of this study related to its retrospective and single-center design, it is important to note that we did not apply the classification published by the RANO Resect Group in 2023.^29^ The extent of resection was classified into three groups: biopsy, partial resection and subtotal or radiological complete resection as defined in Material and Methods. The extent of resection was assessed based on the largest tumor measurement in the three spatial planes.

*MGMT* promoter methylation was found in 39.8% of our patients, consistent with reported rates of 39%-45% in various cohorts, and up to 63% in some clinical trials.^23^^,^^30^ As expected, the presence of *MGMT* promoter methylation clearly appeared to be a favorable prognostic factor for OS and PFS in our cohort.

We have compared the alterations most found in our study with those described in similar works in the literature in the Supplementary Appendix 6 (see online supplementary material). The literature reports an average of 14 alterations per patient during screening, similar to our cohort where the average number was 13.^30^ The proportions in our cohort were largely comparable without a few exceptions, such as a high prevalence of *NOTCH1*, *PTPN11* and *KMT2D* (*MLL2*) substitutions, particularly with unknown mutations.^8^^,^^23^^,^^31^

Moreover, 3.6% of our population exhibited a high *de novo* TMB which is relatively close to the 5% reported in three literature-based databases (DFCI-Profile; MSKCC-IMPACT; FMI) including glioma. It should be noted that these databases include *IDH*-mutant cases.^32^

In our study, and to the best of our knowledge for the first time, an increase in the number of alterations within *IDH*wt GBM was significantly associated with a poor prognosis in OS. However, TMB remained low within for 96.4% of our cohort. The association between decreased OS and increased CNAs had already been described in glioma.^33^

In our work, the loss of *CDKN2A* and/or *CDKN2B* was significantly associated with poorer OS and PFS. This association has been well-documented in *IDH*-mutated glioma, often leading to their reclassification as grade 4 based on this single molecular alteration, even when histology indicates a lower grade. Its role in the prognosis of *IDH*wt GBM has been less clearly defined. However, our findings strengthen this connection.^5^^,^^34^^,^^35^

Surprisingly, *EGFR* substitutions emerged as to be associated with better OS. To our knowledge, this is the first study to report this association.

In GBM, *EGFR* is a potential driver gene with two main alterations studied: amplification (found in nearly 40%-50% of GBM) and *EGFRvIII* constitutively active deletion (in-frame deletion that removes exons 2 to 7 in the extracellular domain of the *EGFR* gene).^31^^,^^36^  *EGFR* amplification was identified as an early event because *EGFRvIII* mutations emerge from intratumoral heterogeneity later in tumor development.^37^

All *EGFR* mutations are often associated with amplified *EGFR* but they can also be detected without amplification.^36^^,^^38^^,^^39^ In our study, 24% presented an *EGFR* substitution without *EGFR* amplification, as previously described.

It is important to note that the FMI test used in our study does not allow us to distinguish the in-frame *EGFRvIII* deletion from other heterozygous deletions of *EGFR*.

Several substitutions have been identified in *EGFR* in GBM, primarily in the extracellular domain, in contrast to non-small-cell lung cancer, where *EGFR* mutations are more commonly found in the intracellular tyrosine kinase domain (Supplementary Appendix 7—see online supplementary material).^36^^,^^40^

The prognostic role of *EGFR* alterations has not been clearly established to date. Moreover, inhibition of *EGFR* has proven to be an ineffective clinical strategy for GBM.^41^ While interesting preclinical results had been reported, numerous phase II and III trials of Tyrosine kinase inhibitors targeting *EGFR* did not present a clear clinical and survival benefit.^42^^,^^43^

Only one study, published by Binder et al. in *Cancer Cell*, demonstrated a decrease in survival associated with the presence of a missense mutation at codon 289 of *EGFR,* which is distinct from *EGFR* amplification and *MGMT* methylation status. There was no impact on survival for the other two oncogenic missense mutations studied (R108 and G598).^44^

In subgroup analyses, patients with A289 or G598 mutations showed shorter median OS than the overall cohort, but the small sample size precluded multivariable adjustment. Despite the poor prognosis associated with A289, the observed link between *EGFR* substitutions and improved OS suggests underlying biological heterogeneity. The presence of VUS also raises questions about their functional impact (neutral or pathogenic), warranting validation in external cohorts and mechanistic studies in model systems.

Furthermore, in 2022, a comprehensive study published in *Nature* examined the various alterations found in *EGFR* in GBM and their pathogenic implications. The study concluded that the aberrant forms of *EGFR* found in GBM seem to exhibit limited activity. *EGFRvIII* variant appeared only weakly activated, and most of single amino acid substitutions observed in GBM did not lead to significant ligand-independent constitutive activation. Unlike in lung cancer, where *EGFR* mutations directly promote EGFR kinase activity, it is suggested that *EGFR* abnormalities in GBM may instead alter the qualitative nature of *EGFR* signaling. For instance, these aberrations could influence signaling to the tumor microenvironment.^45^

*EGFR* substitutions were rarely isolated, often co-occurring with other *EGFR* alterations, suggesting that such combinations may attenuate or inactivate the primary signaling pathway.

Our study also suggested that *TP53* substitutions were associated with shorter OS.

*TP53* mutations are present across various types of cancer, including myeloid and lymphoid malignancies, where they are generally linked to a poor prognosis.

In glial tumors, *TP53* mutations are most seen in low-grade glioma and in grade 4 astrocytoma *IDH* mutant. In 1998, Tohma Y et at. described *TP53* early alterations characteristic of the pathway leading to secondary GBM, were frequent in these tumors (∼25%-65%) but much rarer in primary GBM (<15%).^46^ Unlike the frequently multiple *EGFR* alterations, *TP53* mutations in our cohort were typically single. Most identified *TP53* substitutions have been previously reported in other cancers and are commonly associated with oncogenic functions (Supplementary Appendix 8—see online supplementary material).

In GBM, various studies, mostly conducted on small cohorts, suggested a contradictory prognostic role of *TP53* mutations.^47^^,^^48^ However, in 2023, Kessler et al. found a significantly higher prevalence of *TP53* mutations in patients with the lowest OS.^8^

Several hypotheses support an unfavorable prognostic role for *TP53* substitutions. Wang et al. reported an association between *TP53* mutations and poor OS in GBM, with mutations in exons 4-8 reducing TMZ sensitivity by increasing *MGMT* expression.^49^ Other proposed mechanisms include INTS9 overexpression, linked to *TP53* mutations and enhanced proliferation, and *TP53*-driven upregulation of AUP1, potentially promoting inflammatory microenvironments in GBM.^50^^,^^51^

*NOTCH1* substitutions were found in a notable subset of GBM and showed a favorable PFS trend, though all were variants of uncertain significance (Supplementary Appendix 9—see online supplementary material). Their biological impact remains unclear, but the recurrent R912W mutation merits further study in larger cohorts and preclinical models.^52^

## Conclusion

For the first time, our study highlights the favorable prognostic role of *EGFR* substitutions in *IDH*wt GBM, after adjustment on age, number of alterations, *MGMT* promoter methylation status, performance status, the extent of surgical resection, the tumor localization and the MIB1. Conversely, *TP53* substitutions and *CDKN2A/B* loss were associated with poor prognostic in OS. The presence of a *NOTCH1* substitution appeared to show a trend towards a better prognosis in PFS.

The prognostic impact of these new molecular alterations in *IDH*wt GBM needs to be validated in external cohorts, and if confirmed, additional studies should be conducted to explore their pathophysiological role in cell and mouse models.

## Supplementary Material

oyag095_Supplementary_Data

## Data Availability

Health Data Hub (MR004) approved the study (no. 20823393).

## References

[1] Louis DN , Perry A, Reifenberger G, et al The 2016 World Health Organization Classification of Tumors of the Central Nervous System: a summary. Acta Neuropathol. 2016;131:803-820. 10.1007/s00401-016-1545-1 27157931

[2] Brat DJ , Aldape K, Colman H, et al cIMPACT-NOW update 3: recommended diagnostic criteria for ‘Diffuse astrocytic glioma, IDH-wildtype, with molecular features of glioblastoma, WHO grade IV’. Acta Neuropathol. 2018;136:805-810. 10.1007/s00401-018-1913-0 30259105 PMC6204285

[3] Louis DN , Perry A, Wesseling P, et al The 2021 WHO Classification of Tumors of the Central Nervous System: a summary. Neuro-Oncol. 2021;23:1231-1251. 10.1093/neuonc/noab106 34185076 PMC8328013

[4] Ma S , Rudra S, Campian JL, et al Prognostic impact of CDKN2A/B deletion, TERT mutation, and EGFR amplification on histological and molecular IDH-wildtype glioblastoma. Neurooncol Adv. 2020;2:vdaa126. 10.1093/noajnl/vdaa126 33235995 PMC7668466

[5] Liu EM , et al Molecular landscape of IDH-wild type, pTERT-wild type adult glioblastomas. Brain Pathol. Zurich Switz. 2022;32:e13107. 10.1111/bpa.13107 PMC9616088 35815721

[6] Stupp R , Mason WP, van den Bent MJ, et al Radiotherapy plus concomitant and adjuvant temozolomide for glioblastoma. N Engl J Med. 2005;352:987-996. 10.1056/NEJMoa043330 15758009

[7] Stupp R , Taillibert S, Kanner A, et al Effect of tumor-treating fields plus maintenance temozolomide vs maintenance temozolomide alone on survival in patients with glioblastoma: a randomized clinical trial. JAMA. 2017;318:2306-2316. 10.1001/jama.2017.18718 29260225 PMC5820703

[8] Kessler T , Schrimpf D, Doerner L, et al Prognostic markers of DNA methylation and next-generation sequencing in progressive glioblastoma from the EORTC-26101 trial. Clin Cancer Res. 2023;29:3892-3900. 10.1158/1078-0432.CCR-23-0926 37494539 PMC10543963

[9] Long GV , Flaherty KT, Stroyakovskiy D, et al Dabrafenib plus trametinib versus dabrafenib monotherapy in patients with metastatic BRAF V600E/K-mutant melanoma: long-term survival and safety analysis of a phase 3 study. Ann Oncol. 2019;30:1848. 10.1093/annonc/mdz221 31406976 PMC6927319

[10] Riely GJ , Wood DE, Ettinger DS, et al Non-small cell lung cancer, Version 4.2024, NCCN Clinical Practice Guidelines in Oncology. J Natl Compr Canc Netw. 2024;22:249-274. 10.6004/jnccn.2204.0023 38754467

[11] Le Rhun E , Preusser M, Roth P, et al Molecular targeted therapy of glioblastoma. Cancer Treat Rev. 2019;80:101896. 10.1016/j.ctrv.2019.101896 31541850

[12] Wen PY , Stein A, van den Bent M, et al Dabrafenib plus trametinib in patients with BRAFV600E-mutant low-grade and high-grade glioma (ROAR): a multicentre, open-label, single-arm, phase 2, basket trial. Lancet Oncol. 2022;23:53-64. 10.1016/S1470-2045(21)00578-7 34838156

[13] Wen PY , Touat M, Alexander BM, et al Buparlisib in patients with recurrent glioblastoma harboring phosphatidylinositol 3-kinase pathway activation: an open-label, multicenter, multi-arm, phase II trial. J Clin Oncol. 2019;37:741-750. 10.1200/JCO.18.01207 30715997 PMC6553812

[14] Ma DJ , Galanis E, Anderson SK, et al A phase II trial of everolimus, temozolomide, and radiotherapy in patients with newly diagnosed glioblastoma: NCCTG N057K. Neuro Oncol. 2015;17:1261-1269. 10.1093/neuonc/nou328 25526733 PMC4588750

[15] Taylor JW , Parikh M, Phillips JJ, et al Phase-2 trial of palbociclib in adult patients with recurrent RB1-positive glioblastoma. J Neurooncol. 2018;140:477-483. 10.1007/s11060-018-2977-3 30151703 PMC6239922

[16] Cloughesy TF , Drappatz J, de Groot J, et al Phase II study of cabozantinib in patients with progressive glioblastoma: subset analysis of patients with prior antiangiogenic therapy. Neuro-Oncol. 2018;20:259-267. 10.1093/neuonc/nox151 29036345 PMC5777491

[17] Frampton GM , Fichtenholtz A, Otto GA, et al Development and validation of a clinical cancer genomic profiling test based on massively parallel DNA sequencing. Nat. Biotechnol. 2013;31:1023-1031. 10.1038/nbt.2696 24142049 PMC5710001

[18] Perry JR , Laperriere N, O’Callaghan CJ, et al Short-Course radiation plus temozolomide in elderly patients with glioblastoma. N Engl J Med. 2017;376:1027-1037. 10.1056/NEJMoa1611977 28296618

[19] Chinot OL , Wick W, Mason W, et al Bevacizumab plus radiotherapy-temozolomide for newly diagnosed glioblastoma. N Engl J Med. 2014;370:709-722. 10.1056/NEJMoa1308345 24552318

[20] Wang Z , Gao L, Wang Y, et al Validation of the prognostic value of 9-gene ATE score for IDH wild-type glioblastoma. Neuro Oncol. 2021;23:1197-1199. 10.1093/neuonc/noab058 33982756 PMC8248834

[21] Stupp R , Hegi ME, Mason WP, et al Effects of radiotherapy with concomitant and adjuvant temozolomide versus radiotherapy alone on survival in glioblastoma in a randomised phase III study: 5-year analysis of the EORTC-NCIC trial. Lancet Oncol. 2009;10:459-466. 10.1016/S1470-2045(09)70025-7 19269895

[22] Skaga E , Skretteberg MA, Johannesen TB, et al Real-world validity of randomized controlled phase III trials in newly diagnosed glioblastoma: to whom do the results of the trials apply? Neurooncol Adv. 2021;3:vdab008. 10.1093/noajnl/vdab008 33665615 PMC7914075

[23] Lim-Fat MJ , Youssef GC, Touat M, et al Clinical utility of targeted next-generation sequencing assay in IDH-wildtype glioblastoma for therapy decision-making. Neuro Oncol. 2022;24:1140-1149. 10.1093/neuonc/noab282 34878541 PMC9248387

[24] Lacroix M , Abi-Said D, Fourney DR, et al A multivariate analysis of 416 patients with glioblastoma multiforme: prognosis, extent of resection, and survival. J Neurosurg. 2001;95:190-198. 10.3171/jns.2001.95.2.0190 11780887

[25] Tortosa A , Viñolas N, Villà S, et al Prognostic implication of clinical, radiologic, and pathologic features in patients with anaplastic gliomas. Cancer. 2003;97:1063-1071. 10.1002/cncr.11120 12569607

[26] Frappaz D , Bonneville-Levard A, Ricard D, et al Assessment of Karnofsky (KPS) and WHO (WHO-PS) performance scores in brain tumour patients: the role of clinician bias. Support Care Cancer. 2021;29:1883-1891. 10.1007/s00520-020-05663-y 32789684

[27] Hottinger AF , Yoon H, DeAngelis LM, Abrey LE. Neurological outcome of long-term glioblastoma survivors. J Neurooncol. 2009;95:301-305. 10.1007/s11060-009-9946-9 19557499

[28] Hertler C , Felsberg J, Gramatzki D, et al Long-term survival with IDH wildtype glioblastoma: first results from the ETERNITY Brain Tumor Funders’ Collaborative Consortium (EORTC 1419). Eur J Cancer. 2023;189:112913. 10.1016/j.ejca.2023.05.002 37277265

[29] Karschnia P , Young JS, Dono A, et al Prognostic validation of a new classification system for extent of resection in glioblastoma: a report of the RANO resect group. Neuro Oncol. 2023;25:940-954. 10.1093/neuonc/noac193 35961053 PMC10158281

[30] Draaisma K , Chatzipli A, Taphoorn M, et al Molecular evolution of *IDH* wild-type glioblastomas treated with standard of care affects survival and design of precision medicine trials: a report from the EORTC 1542 study. J Clin Oncol. 2020;38:81-99. 10.1200/JCO.19.00367 31743054

[31] Padovan M , Maccari M, Bosio A, et al Next-generation sequencing (NGS) for identifying actionable molecular alterations in patients with newly diagnosed and recurrent IDHwt-glioblastoma (GBM): a large mono-institutional experience. JCO. 2022;40:3139-3139. 10.1200/JCO.2022.40.16_suppl.3139 37481865

[32] Touat M , Li YY, Boynton AN, et al Mechanisms and therapeutic implications of hypermutation in gliomas. Nature. 2020;580:517-523. 10.1038/s41586-020-2209-9 32322066 PMC8235024

[33] Chen C-H , Lin Y-J, Lin Y-Y, et al Glioblastoma primary cells retain the most copy number alterations that predict poor survival in glioma patients. Front Oncol. 2021;11:621432. 10.3389/fonc.2021.621432 33981597 PMC8108987

[34] Appay R , Dehais C, Maurage C-A, et al CDKN2A homozygous deletion is a strong adverse prognosis factor in diffuse malignant IDH-mutant gliomas. Neuro Oncol. 2019;21:1519-1528. 10.1093/neuonc/noz124 31832685 PMC7145561

[35] Hsu EJ , Thomas J, Maher EA, et al Impact of CDKN2A/B, MTAP, and TERT genetic alterations on survival in IDH wild type glioblastomas. Discov Oncol. 2022;13:126. 10.1007/s12672-022-00590-2 36380219 PMC9666584

[36] An Z , Aksoy O, Zheng T, Fan Q-W, Weiss WA. Epidermal growth factor receptor and EGFRvIII in glioblastoma: signaling pathways and targeted therapies. Oncogene. 2018;37:1561-1575. 10.1038/s41388-017-0045-7 29321659 PMC5860944

[37] Eskilsson E , Rosland GV, Talasila KM, et al EGFRvIII mutations can emerge as late and heterogenous events in glioblastoma development and promote angiogenesis through Src activation. Neuro Oncol. 2016;18:1644-1655. 10.1093/neuonc/now113 27286795 PMC5791772

[38] Brennan CW , Verhaak RGW, McKenna A, et al The somatic genomic landscape of glioblastoma. Cell. 2013;155:462-477. 10.1016/j.cell.2013.09.034 24120142 PMC3910500

[39] Felsberg J , Hentschel B, Kaulich K, et al Epidermal growth factor receptor variant III (EGFRvIII) positivity in *EGFR*-amplified glioblastomas: Prognostic role and comparison between primary and recurrent tumors. Clin Cancer Res. 2017;23:6846-6855. 10.1158/1078-0432.CCR-17-0890 28855349

[40] Sharma SV , Bell DW, Settleman J, Haber DA. Epidermal growth factor receptor mutations in lung cancer. Nat Rev Cancer. 2007;7:169-181. 10.1038/nrc2088 17318210

[41] Dewdney B , Jenkins MR, Best SA, et al From signalling pathways to targeted therapies: unravelling glioblastoma’s secrets and harnessing two decades of progress. Signal Transduct Target Ther. 2023;8:400. 10.1038/s41392-023-01637-8 37857607 PMC10587102

[42] Schulte A , Liffers K, Kathagen A, et al Erlotinib resistance in EGFR-amplified glioblastoma cells is associated with upregulation of EGFRvIII and PI3Kp110. Neuro Oncol. 2013;15:1289-1301. 10.1093/neuonc/not093 23877316 PMC3779041

[43] Westphal M , Maire CL, Lamszus K. EGFR as a target for glioblastoma treatment: an unfulfilled promise. CNS Drugs. 2017;31:723-735. 10.1007/s40263-017-0456-6 28791656 PMC5573763

[44] Binder ZA , Thorne AH, Bakas S, et al Epidermal growth factor receptor extracellular domain mutations in glioblastoma present opportunities for clinical imaging and therapeutic development. Cancer Cell. 2018;34:163-177.e7. 10.1016/j.ccell.2018.06.006 29990498 PMC6424337

[45] Hu C , Leche CA, Kiyatkin A, et al Glioblastoma mutations alter EGFR dimer structure to prevent ligand bias. Nature. 2022;602:518-522. 10.1038/s41586-021-04393-3 35140400 PMC8857055

[46] Tohma Y , Gratas C, Biernat W, et al PTEN (MMAC1) mutations are frequent in primary glioblastomas (de novo) but not in secondary glioblastomas. J Neuropathol Exp Neurol. 1998;57:684-689. 10.1097/00005072-199807000-00005 9690672

[47] Krex D , Klink B, Hartmann C, et al Long-term survival with glioblastoma multiforme. Brain. 2007;130:2596-2606. 10.1093/brain/awm204 17785346

[48] Yan Y , Takayasu T, Hines G, et al Landscape of genomic alterations in *IDH* wild-type glioblastoma identifies PI3K as a favorable prognostic factor. JCO Precis. Oncol. 2020;4:575-584. 10.1200/PO.19.00385 35050747

[49] Wang X , Chen J-X, Liu J-P, et al Gain of function of mutant TP53 in glioblastoma: prognosis and response to temozolomide. Ann Surg Oncol. 2014;21:1337-1344. 10.1245/s10434-013-3380-0 24248532

[50] Lin Y-C , Chang P-C, Hueng D-Y, Huang S-M, Li Y-F. Decoding the prognostic significance of integrator complex subunit 9 (INTS9) in glioma: links to TP53 mutations, E2F signaling, and inflammatory microenvironments. Cancer Cell Int. 2023;23:154. 10.1186/s12935-023-03006-5 37537630 PMC10401760

[51] Chang P-C , Lin Y-C, Yen H-J, et al Ancient ubiquitous protein 1 (AUP1) is a prognostic biomarker connected with TP53 mutation and the inflamed microenvironments in glioma. Cancer Cell Int. 2023;23:62. 10.1186/s12935-023-02912-y 37029364 PMC10080956

[52] Agrawal N , Frederick MJ, Pickering CR, et al Exome sequencing of head and neck squamous cell carcinoma reveals inactivating mutations in NOTCH1. Science. 2011;333:1154-1157. 10.1126/science.1206923 21798897 PMC3162986

