# Supplementary material for: Identification of new prognostic molecular markers in glioblastoma: a single-center retrospective study
Source: Oncologist. 2026 Mar 16;31(5):oyag095. doi: 10.1093/oncolo/oyag095 (PMC13070693; doi:10.1093/oncolo/oyag095)
Supplement: oyag095_Supplementary_Data [file oyag095_supplementary_data.zip › Supplementary Data ARTICLE 02272026.docx]

Supplementary Data

**Supplementary Appendix 1. Performance status : WHO/ECOG score**

**
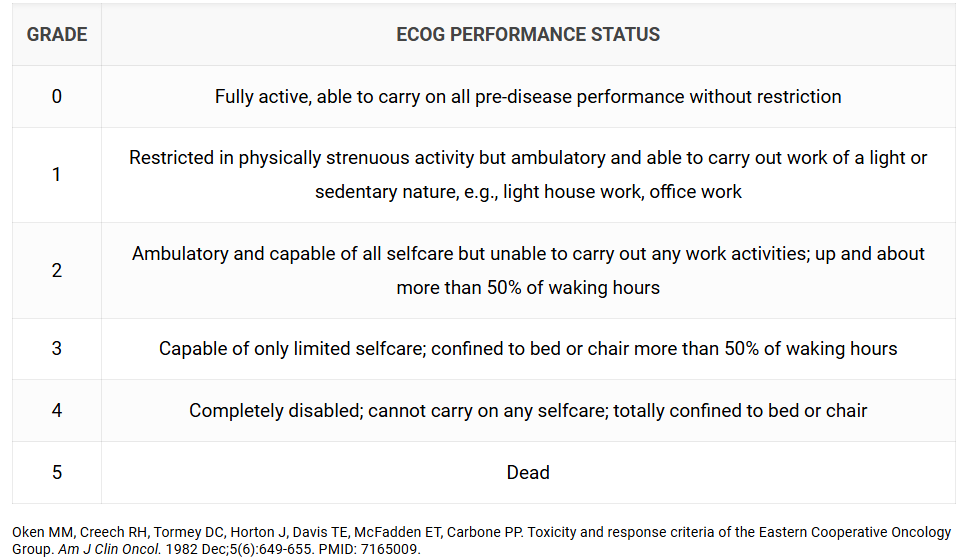
**

**Supplementary Appendix 2. Targeted genes by FMI**

F1CDxTechnical_Specifications_Commercial_SPEC-01197_V3.0.pdf (foundationmedicine.com)


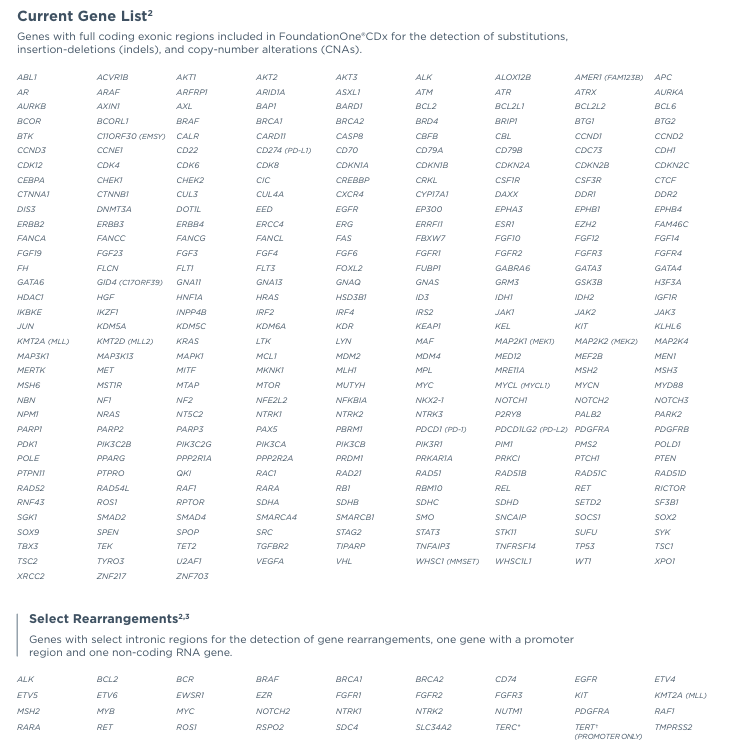


**Supplementary Appendix 3. Frequency of Molecular Alteration in Select Genes in Cohort of patients**

| **Gene** | **Type of alteration** | **Total n = 184** | **%** |
| --- | --- | --- | --- |
| *TERT* promoter | Substitution | 163 | 88.5 |
| *CDKN2A* | Loss | 121 | 65.8 |
| *CDKN2B* | Loss | 113 | 61.4 |
| *MTAP* | Loss | 95 | 51.6 |
| *EGFR* | Amplification | 86 | 46.7 |
|  | Heterozygous deletion | 57 | 31 |
|  | Fusion | 19 | 10.3 |
|  | Substitution | 53 | 28.8 |
| *PTEN* | Loss | 71 | 38.6 |
|  | Codon stop | 18 | 9.8 |
|  | Frameshift | 14 | 7.6 |
|  | Substitution | 26 | 15.2 |
| *TP53* | Substitution | 39 | 21.2 |
|  | Codon stop | 10 | 5.4 |
|  | Splice site | 8 | 4.3 |
| *KMT2D (MLL2)* | Substitution | 24 | 13 |
| *NOTCH1* | Substitution | 21 | 11.4 |
| *CDK4* | Amplification | 20 | 10.9 |
| *MDM4* | Amplification | 20 | 10.9 |
| *NF1* | Substitution | 19 | 10,3 |
|  | Frameshift | 15 | 8.2 |
|  | Codon stop | 11 | 6 |
| *PIK3C2B* | Amplification | 19 | 10.3 |
|  | Substitution | 7 | 3.8 |
| *PTPN11* | Substitution | 17 | 9.2 |
| *PDGFRA* | Amplification | 16 | 8.7 |
|  | Substitution | 15 | 8.2 |
| *ATM* | Substitution | 16 | 8.7 |
| *PIK3CA* | Amplification | 16 | 8.7 |
| *MAP3K1* | Substitution | 15 | 8.2 |

*The number of alterations reported in the Appendix 4. refers to the number of patients presenting with it at least once.*

**Supplementary Appendix 4. OS (A) and PFS (B) univariable analyses : gene / alteration (n = 161)**

**A.**

| **Event** | **HR [95%CI]** | **p-value** |
| --- | --- | --- |
| ***CDKN2A/CDKN2B* - Loss** | | |
| No | 1.00 |  |
| Yes | 1.08 [1.33 ; 3.22] | 0.653 |
| ***EGFR* - Substitution** | | |
| No | 1.00 |  |
| Yes | 0.60 [0.41 ; 0.88] | **0.009** |
| ***KMT2D (MLL2)* - Substitution** | | |
| No | 1.00 |  |
| Yes | 1.51 [0.89 ; 2.56] | 0.128 |
| ***PTEN* - Loss** | | |
| No | 1.00 |  |
| Yes | 0.72 [0.51 ; 1.02] | *0.067* |
| ***PTEN* - Substitution** | | |
| No | 1.00 |  |
| Yes | 1.31 [0.83 ; 2.06] | 0.239 |
| ***TP53* - Substitution** | | |
| No | 1.00 |  |
| Yes | 1.27 [0.85 ; 1.89] | 0.245 |
| ***PTPN11 - Substitution*** | | |
| No | 1.00 |  |
| Yes | 0.84 [0.47 ; 1.53] | 0.997 |
| ***NOTCH1* - Substitution** | | |
| No | 1.00 |  |
| Yes | 1.20 [0.70 ; 2.05] | 0.511 |
| ***CDK4* - Amplification** | | |
| No | 1.00 |  |
| Yes | 1.52 [0.89 ; 2.57] | 0.123 |
| ***PIK3KCA* - Substitution** | | |
| No | 1.00 |  |
| Yes | 0.83 [0.45 ; 1.53] | 0.546 |
| ***MGMT* promoter methylation status (%)** | | |
| Unmethylated | 1.00 |  |
| Methylated | 0.41 [0.28 ; 0.60] | **< 0.001** |
| **MIB1 (%)** | 1.01 [1.00 ; 1.02] | **0.022** |
| **Number of alterations** | 1.01 [1.00 ; 1.02] | 0.128 |

**B.**

| **Event** | **HR [95%CI]** | **p-value** |
| --- | --- | --- |
| ***CDKN2A/CDKN2B* - Loss** | | |
| No | 1.00 |  |
| Yes | 1.25 [0.89 ; 1.74] | 0.193 |
| ***EGFR* - Substitution** | | |
| No | 1.00 |  |
| Yes | 0.83 [0.59 ; 1.16] | 0.274 |
| ***KMT2D (MLL2)* - Substitution** | | |
| No | 1.00 |  |
| Yes | 1.27 [0.76 ; 2.11] | 0.360 |
| ***PTEN* - Loss** | | |
| No | 1.00 |  |
| Yes | 0.80 [0.58 ; 1.11] | 0.184 |
| ***PTEN* - Substitution** | | |
| No | 1.00 |  |
| Yes | 1.34 [0.88 ; 2.03] | 0.172 |
| ***TP53* - Substitution** | | |
| No | 1.00 |  |
| Yes | 0.95 [0.65 ; 1.40] | 0.811 |
| ***PTPN11 -* Substitution** | | |
| No | 1.00 |  |
| Yes | 0.80 [0.45 ; 1.42] | 0.439 |
| ***NOTCH1* - Substitution** | | |
| No | 1.00 |  |
| Yes | 0.87 [0.52 ; 1.46] | 0.599 |
| ***CDK4* - Amplification** | | |
| No | 1.00 |  |
| Yes | 1.58 [0.94 ; 2.65] | *0.082* |
| ***PIK3KCA* - Substitution** | | |
| No | 1.00 |  |
| Yes | 1.44 [0.83 ; 2.50] | 0.198 |
| ***MGMT* promoter methylation status (%)** | | |
| Unmethylated | 1.00 |  |
| Methylated | 0.40 [0.28 ; 0.57] | **< 0.001** |
| **MIB1 (%)** | 1.01 [1.00 ; 1.02] | 0.321 |
| **Number of alterations** | 1.01 [1.00 ; 1.02] | 0.269 |

**Supplementary Appendix 5. OS (A) and PFS (B) according to Landmark at 2 months analysis (n = 119)**

**A.**

| **Event** | **HR [95%CI]** | **p-value** |
| --- | --- | --- |
| ***CDKN2A/CDKN2B* - Loss** | | |
| No | 1.00 |  |
| Yes | 1.62 [0.96 ; 2.75] | *0.072* |
| ***EGFR* – Substitution** | | |
| No | 1.00 |  |
| Yes | 0. 34 [0.33 ; 0.85] | **< 0.001** |
| ***KMT2D (MLL2)* - Substitution** | | |
| No | 1.00 |  |
| Yes | 1.36 [0.70 ; 2.66] | 0.367 |
| ***PTEN* – Substitution** | | |
| No | 1.00 |  |
| Yes | 0.75 [0.40 ; 1.40] | 0.367 |
| ***TP53* - Substitution** | | |
| No | 1.00 |  |
| Yes | 1.53 [0.89 ; 2.64] | 0.124 |
| **Age at diagnosis** | | |
| < 60 | 1.00 |  |
| >= 60 | 1.60 [1.00 ; 2.57] | **0.049** |
| ***Performance* status** | | |
| 0-1 | 1.00 |  |
| 2-3 | 1.07 [0.59 ; 1.94] | 0.828 |
| **Extent of resection** | | |
| Biopsy | 1.00 |  |
| Partial resection | 0.53 [0.28 ; 1.00] | **0.049** |
| Subtotal / macroscopically complete | 0.41 [0.21 ; 0.81] | **0.011** |
| ***MGMT* promoter methylation status (%)** | | |
| Unmethylated | 1.00 |  |
| Methylated | 0.37 [0.21 ; 0.61] | **< 0.001** |
| **Tumor localization** | | |
| Other | 1.00 |  |
| Frontal | 1.15 [0.73 ; 1.81] | 0.542 |
| **Number of alterations** | 1.10 [1.03 ; 1.19] | **0.008** |
| **MIB1 (%)** | 1.01 [1.00 ; 1.03] | 0.136 |

**B.**

| **Event** | **HR [95%CI]** | **p-value** |
| --- | --- | --- |
| ***CDKN2A/CDKN2B* - Loss** | | |
| No | 1.00 |  |
| Yes | 1.60 [0.98 ; 2.60] | *0.059* |
| ***KMT2D (MLL2)* - Substitution** | | |
| No | 1.00 |  |
| Yes | 1.37 [0.71 ; 2.63] | 0.350 |
| ***NOTCH1* – Substitution** | | |
| No | 1.00 |  |
| Yes | 0.51 [0.25 ; 1.03] | *0.062* |
| ***PIK3KCA* - Substitution** | | |
| No | 1.00 |  |
| Yes | 1.71 [0.85 ; 3.44] | 0.132 |
| ***PTEN* - Substitution** | | |
| No | 1.00 |  |
| Yes | 1.09 [0.63 ; 1.88] | 0.750 |
| **Age at diagnosis** | | |
| < 60 | 1.00 |  |
| >= 60 | 1.30 [0.84; 2.01] | 0.236 |
| ***Performance* status** | | |
| 0-1 | 1.00 |  |
| 2-3 | 0.95 [0.56 ; 1.60] | 0.835 |
| **Extent of resection** | | |
| Biopsy | 1.00 |  |
| Partial resection | 0.94 [0.53 ; 1.67] | 0.837 |
| Subtotal / macroscopically complete | 1.05 [0.56 ; 1.99] | 0.874 |
| ***MGMT* promoter methylation status (%)** | | |
| Unmethylated | 1.00 |  |
| Methylated | 0.23 [0.14 ; 0.37] | **< 0.001** |
| **Tumor localization** | | |
| Other | 1.00 |  |
| Frontal | 1.18 [0.78 ; 1.79] | 0.434 |
| **Number of alterations** | 1.05 [0.99 ; 1.12] | 0.121 |
| **MIB1 (%)** | 1.01 [1.00 ; 1.03] | 0.101 |

**Supplementary Appendix 6. Comparison of the rate of occurrence of the molecular alterations most frequently found in molecular screening during the diagnosis of GBM between our study and the literature.**

| **Gene** | **Type of alteration** | **Total**  **n = 184** | **Us (%)** | **Lim Fat, *Neuro oncol*. 2022 (%)** | **Kessler, *CCR.* 2023 (%)** | **Padovan, *EJC.* 2022 (%)** |
| --- | --- | --- | --- | --- | --- | --- |
| TERT prom. | Substitution | 163 | 88.5 | 63.6 |  | 84.6 |
| CDKN2A | Loss | 121 | 65.8 | 45.6 |  | 53.2 |
| PTEN | Loss | 71 | 38.6 | 35.2 |  | 45 |
|  | Substitution | 26 | 15.2 |  | 23 | 35.5 |
| CDKN2B | Loss | 113 | 61.4 | 41.4 |  | 50.7 |
| EGFR | Amplification | 86 | 46.7 | 33.4 |  | 38.3 |
|  | Substitution | 53 | 28.8 |  | 14 | 25.6 |
|  | Heterozygous deletion | 57 | 31* |  |  |  |
| MTAP | Loss | 95 | 51.6 | 38.2 |  |  |
| TP53 | Substitution | 39 | 21.2 | 28.9 | 17 |  |
| NF1 | Substitution | 19 | 10.3 | 9.7 | 9 | 18.7 |
| PDGFRA | Amplification | 16 | 8.7 | 7.7 |  | 7.9 |
| PIK3CA/2B | Amplification | 35 | 19 | 5.6 |  | 16.5 |
| KMT2D (*MLL2)* | Substitution | 24 | 13 |  | 8 |  |
| NOTCH1 | Substitution | 21 | 11.4 | 0.5 |  |  |
| CDK4 | Amplification | 20 | 10.9 | 11 |  | 16.2 |
| MDM4 | Amplification | 20 | 10.9 | 5.6 |  |  |
| PTPN11 | Substitution | 17 | 9.2 | 1.3 |  |  |
| POLE | Substitution | 11 | 6 | 0.7 |  | 7.4 |
| BRCA 1/2 | Substitution | 19 | 10.3 |  |  | 7.9 |
| BRAF V600E | Substitution | 8 | 4.3 | 2.2 |  | 2.5 |
| RB1 |  | 22 | 12 | 7.4 |  | 13.2 |

** Heterozygous deletion not reported in the studies presented above. It should be noted that the EGFRvIII variant was found in 25 to 30% of patients in different cohorts from the literature*^50,51^.

**Supplementary Appendix 7. EGFR Substitutions reported and compared to data from literature (OncoKB)**

| *EGFR* | | | | |
| --- | --- | --- | --- | --- |
| VARIATION | NUMBER OF PATIENTS | ROLE | LOCALIZATION | DOMAIN |
| S768I | 2 | ONCOGENIC | LUNG | ICR |
| G598V | 10 | ONCOGENIC | GBM | ECR |
| L861Q | 1 | ONCOGENIC | LUNG |  |
| R108K | 7 | ONCOGENIC | LUNG  (*IN VITRO* GBM) | ECR |
| V774M | 1 | POSSIBLE | *IN VITRO* |  |
| T790M | 1 | ONCOGENIC | LUNG | ICR |
| R324L | 2 | ONCOGENIC | GBM | ECR |
| T263P | 2 | ONCOGENIC | GBM | ECR |
| A289V | 7 | ONCOGENIC | GBM | ECR |
| A289F | 1 | UNKNOWN |  |  |
| A289T | 3 | POSSIBLE | *IN VITRO* |  |
| R222C | 4 | POSSIBLE | *IN VITRO* | ECR |
| Y610C | 1 | UNKNOWN |  |  |
| A597P | 1 | UNKNOWN |  |  |
| L62R | 1 | POSSIBLE | *IN VITRO* | ECR |
| P596L | 5 | POSSIBLE | *IN VITRO* | ECR |
| D46N | 1 | UNKNOWN |  |  |
| E45K | 1 | UNKNOWN |  |  |
| R494G | 1 | UNKNOWN |  |  |
| A289D | 1 | POSSIBLE | *IN VITRO* | ECR |
| G665D | 1 | UNKNOWN |  |  |
| S645C | 1 | POSSIBLE | *IN VITRO* | ICR |
| D761N | 1 | POSSIBLE | *IN VITRO* | ICR |
| R252C | 1 | POSSIBLE | *IN VITRO* | ECR |
| D256Y | 1 | POSSIBLE | *IN VITRO* | ECR |
| S227F | 2 | UNKNOWN |  |  |
| C311R | 1 | UNKNOWN |  |  |
| C539G | 1 | UNKNOWN |  |  |
| Y764C | 1 | UNKNOWN |  |  |
| R677H | 1 | UNKNOWN |  |  |
| T572R | 1 | UNKNOWN |  |  |
| K716E | 1 | UNKNOWN |  |  |
| G331R | 1 | UNKNOWN |  |  |
| G63R | 1 | POSSIBLE | *IN VITRO* | ECR |
| V651E | 1 | UNKNOWN |  |  |
| C620Y | 1 | POSSIBLE | *IN VITRO* | ECR |
| Q276L | 1 | UNKNOWN |  |  |
| R324H | 1 | UNKNOWN |  |  |
| *TOTAL* | *72* |  |  |  |

*ICR : intracellular ; ECR : extracellular*

*Each substitution identified in EGFR gene was documented and compared to the existing data in the literature (Data from OncoKB™ - MSK's Precision Oncology Knowledge Base) based on the known oncogenic status, the type of tumor (in vivo or in vitro) and the functional domain.*

**Supplementary Appendix 8. TP53 Substitutions reported and compared to data from literature (OncoKB)**

*Each substitution identified in TP53 gene was documented and compared to the existing data in the literature (Data from OncoKB™ - MSK's Precision Oncology Knowledge Base) based on the known oncogenic status, the function and the type of tumor (in vivo or in vitro).*

*Regarding known alterations, the domain of action is the same : DNA binding domain of the protein*

**Supplementary Appendix 9. NOTCH1 Substitutions reported and compared to data from literature (OncoKB)**

| *NOTCH 1* | | |
| --- | --- | --- |
| VARIATION | PATIENT NUMBER | ROLE |
| N502S | 1 | UNKNOWN |
| R912W | 3 | UNKNOWN |
| K17774R | 1 | UNKNOWN |
| G2262S | 1 | UNKNOWN |
| G248A | 1 | UNKNOWN |
| S1004L | 1 | UNKNOWN |
| D1533N | 1 | UNKNOWN |
| L1980P | 1 | UNKNOWN |
| R2313Q | 1 | UNKNOWN |
| V1739M | 1 | UNKNOWN |
| G1476S | 1 | UNKNOWN |
| G318D | 1 | UNKNOWN |
| V1909I | 1 | UNKNOWN |
| A973T | 1 | UNKNOWN |
| C1284W | 1 | UNKNOWN |
| V1575L | 1 | UNKNOWN |
|  | *18* |  |

*Each substitution identified in NOTCH1 gene was documented and compared to the existing data in the literature (Data from OncoKB™ - MSK's Precision Oncology Knowledge Base) based on the known oncogenic status. Here, all the substitutions found are unknown in the literature. Therefore, we cannot determine the potential activity or its localization by comparing it with other tumor types.*
